# Chromosome-level assembly of the *Rangifer tarandus* genome and validation of cervid and bovid evolution insights

**DOI:** 10.1186/s12864-023-09189-5

**Published:** 2023-03-23

**Authors:** William Poisson, Julien Prunier, Alexandra Carrier, Isabelle Gilbert, Gabriela Mastromonaco, Vicky Albert, Joëlle Taillon, Vincent Bourret, Arnaud Droit, Steeve D. Côté, Claude Robert

**Affiliations:** 1 Département des sciences animales, Faculté des sciences de l’agriculture et de l’alimentation, Université Laval, Québec, QC Canada; 2 Centre de Recherche en Reproduction, Développement et Santé Intergénérationnelle, Québec, QC Canada; 3 Réseau Québécois en reproduction, QC Saint-Hyacinthe, Canada; 4 Département de biochimie, microbiologie et bio-informatique, Faculté des sciences et de génie, Université Laval, Québec, QC Canada; 5 Toronto Zoo, Toronto, ON Canada; 6 Ministère des Forêts, de la Faune et des Parcs du Québec (MFFP), Québec, QC Canada; 7 Département de médecine moléculaire, Faculté de médecine, Université Laval, Québec, QC, Canada; 8 Caribou Ungava, Département de biologie and Centre d’études nordiques, Faculté des sciences et de génie, Université Laval, Québec, QC Canada

**Keywords:** *Rangifer tarandus*, Genome assembly, Scaffold, Chromosome, Karyotype, Fluorescence in situ hybridization, Oligopaint, Idiogram

## Abstract

**Background:**

Genome assembly into chromosomes facilitates several analyses including cytogenetics, genomics and phylogenetics. Despite rapid development in bioinformatics, however, assembly beyond scaffolds remains challenging, especially in species without closely related well-assembled and available reference genomes. So far, four draft genomes of *Rangifer tarandus* (caribou or reindeer, a circumpolar distributed cervid species) have been published, but none with chromosome-level assembly. This emblematic northern species is of high interest in ecological studies and conservation since most populations are declining.

**Results:**

We have designed specific probes based on Oligopaint FISH technology to upgrade the latest published reindeer and caribou chromosome-level genomes. Using this oligonucleotide-based method, we found six mis-assembled scaffolds and physically mapped 68 of the largest scaffolds representing 78% of the most recent *R. tarandus* genome assembly. Combining physical mapping and comparative genomics, it was possible to document chromosomal evolution among Cervidae and closely related bovids.

**Conclusions:**

Our results provide validation for the current chromosome-level genome assembly as well as resources to use chromosome banding in studies of *Rangifer tarandus*.

**Supplementary Information:**

The online version contains supplementary material available at 10.1186/s12864-023-09189-5.

## Background

Genomic polymorphisms and rearrangements tell the tale of speciation and genomic evolution. Meaningful comparative phylogenetic and cytogenetic studies of species and populations require the assembly of chromosomal reference genomes [1, 2]. In non-model species, full sequencing of chromosomes remains rare [3, 4]. A daunting task facing mappers of chromosome scaffolds is the sequencing and positioning of repeat-containing sequences [5]. Although new technologies such as long read sequencing and proximity tagging of DNA fragments are greatly improving the quality of assembly, positioning of super-scaffolds into structured chromosomes remains challenging. Physical mapping on chromosomal spreads is a proven method of complementing bioinformatic-based assembly [6–8].

Chromosomal assembly is focused on one species at a time. However, reference genomes of sufficient quality can serve as templates for other species. Due to its circumpolar distribution and sensitivity to environmental changes [9–11], *Rangifer tarandus* (subfamily Capreolinae, family Cervidae), commonly known as caribou in North America and reindeer in Eurasia [9, 12], is a prime example of a key species monitored for documenting the impacts of climate change and habitat perturbation. North American caribou populations roam in Alaska, Greenland and northern Canada [13, 14]. Reindeer are found over the entire Northern Eurasian continent and on Svalbard [15, 16]. Many North American and Eurasian herds have been recently declining [11, 17–19].

To protect *Rangifer*, accurate genomic information is crucial, particularly for monitoring genetic diversity over time and for forensic purposes [20, 21]. As *Rangifer* taxonomy has recently been questioned [12], this information becomes instrumental to document and study genomic differences between species and subspecies. Several genomic assemblies have been published [20, 22–24] without mapping of entire chromosome. Based on cytogenetic analyses, the *Rangifer tarandus* genome is divided into 33 autosomal acrocentric chromosome pairs, a submetacentric autosome pair, and the sex pair, of which the Y is acrocentric, and the X is metacentric [25]. No karyotypic differences between European (reindeer) and American (caribou) populations are known [26]. Most studies of *R. tarandus* chromosomes go back decades [25–30], and from conventional G and C banding, an idiogram-based classification of chromosomes has been proposed [29, 30]*.* However, *R. tarandus* autosomes are particularly difficult to identify even with the low-band G-banding pattern because they are of very similar size and shape.

Information obtained from genome sequencing has led to the design of specifically targeted DNA probes. Single-locus FISH probes have been used widely for many years to position and visualize gene loci [31–33]. Development of massively paralleled de novo synthesis procedures has allowed vast increases in numbers of targetable loci [34–36]. PCR-synthesized oligonucleotides (oligo), both long [35] and as short as 70 nucleotides [34, 37, 38] are interesting for FISH studies since it allows generation of a renewable and affordable stock of probes that can be used for downstream cytogenetic research. For instance, pools of short synthetic oligo have proven useful for many studies such as chromosome pairing during meiosis [39], karyotyping [40], chromosomal evolution [41], genome architecture [42] and chromatin folding [43]. Recently, it has been used to upgrade the banana (*Musa* spp.*)* genome with 19 libraries of 20,000 45-mers oligo [44].

The present work aimed using Oligopaint FISH probes to map *R. tarandus* genome scaffolds physically onto chromosomes and thereby updating a scaffold-level assembly proposed previously [20]. To our knowledge, this technology has not yet been used to assess scaffold validity or map chromosomes in mammals. The resulting high-quality reference genome should prove useful as a template for studying other species and provide a solid basis for comparative and evolutionary genomics.

## Results

### Probe design

Each Oligopaint FISH probe consisted of a large group of oligos separated by short gaps. Probe sequences were selected using publicly available tools, namely OligoMiner, Ifpd, and the OOD-FISH pipeline [38, 45]. After completing all probe design steps, 255,000 79-mers were selected and distributed in three pools of 91,500 oligo each for massive parallel synthesis. These oligos were distributed in 170 probes covering the 68 largest scaffolds (> 9.5 Mb) of the latest published genome [20], which corresponds to 78% of the assembled genome (2.01 Gb).

**Fig. 1:**
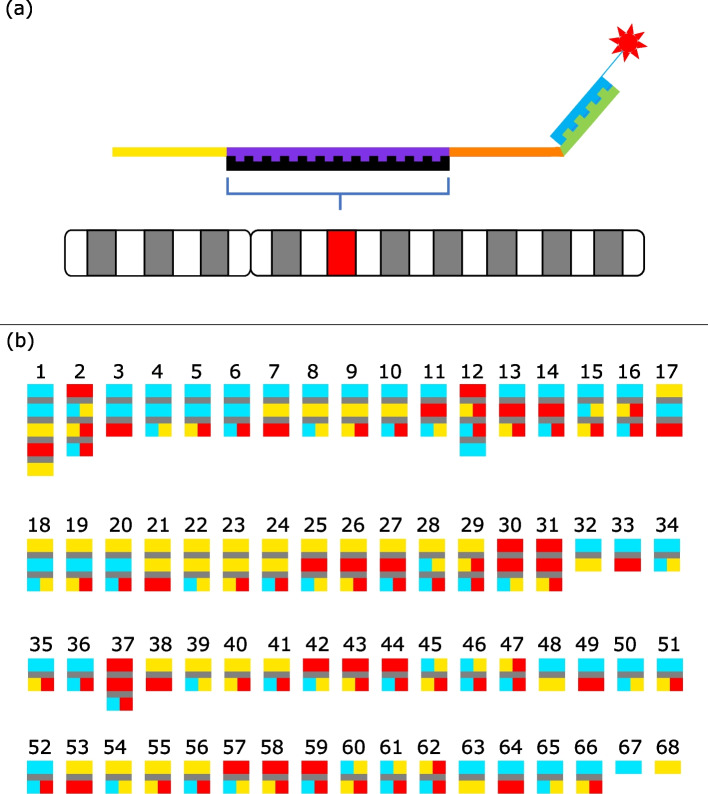
Oligonucleotide’s structure and scaffolds’ color pattern. **a** Oligo comprising a reverse primer sequence (yellow), genome homolog sequence (purple), forward primer sequence (orange) and adapter (green) complementary to the detection oligo (blue) linked to the fluorophore (red). Hybridization of the homolog with its specific genomic complementary sequence (black) is followed by hybridization of the fluorophore-bearing oligo. **b** The 68 scaffolds to be assembled on chromosomes are painted with one to five probes each labeled blue, yellow, or red, giving these three colours or green (blue + yellow), orange (yellow + red) or violet (red + blue). Two-probe patterns were repeated among two or three scaffolds because of limited color possibilities but were hybridized separately to ensure accurate chromosome attribution

Probe distribution had to account for scaffold size and provide a specific colour pattern for identification purposes. For example, a set of five probes was designed for the first and largest scaffold while scaffolds 2 and 12 were addressed with sets of four probes since mis-assembly in the draft genome was suspected. In addition, 29 scaffolds were labelled with sets of three probes, 34 with sets of two probes, and two single probe sets were designed for the last two (shortest) scaffolds. The scaffold colour schemes are detailed in Fig. 1.

Each probe (1500 oligo) spanned on average 401.5 kb of genome (149.4–1121.4 kb, SD: 139.9 kb). Within the same scaffold, probe sets were separated by a mean gap of 12.85 Mb (7.4–24.3 Mb, SD: 3.68 Mb) and located at an average distance of 4.64 Mb (0.25–14.13 Mb, SD: 3.31 Mb) from the scaffold termini. Probe set spacing was highly influenced by sequence quality within the targeted region, where low complexity, high probabilities of dimer or secondary structures and repeated elements could be avoided.

### Chromosome assembly

**Fig. 2:**
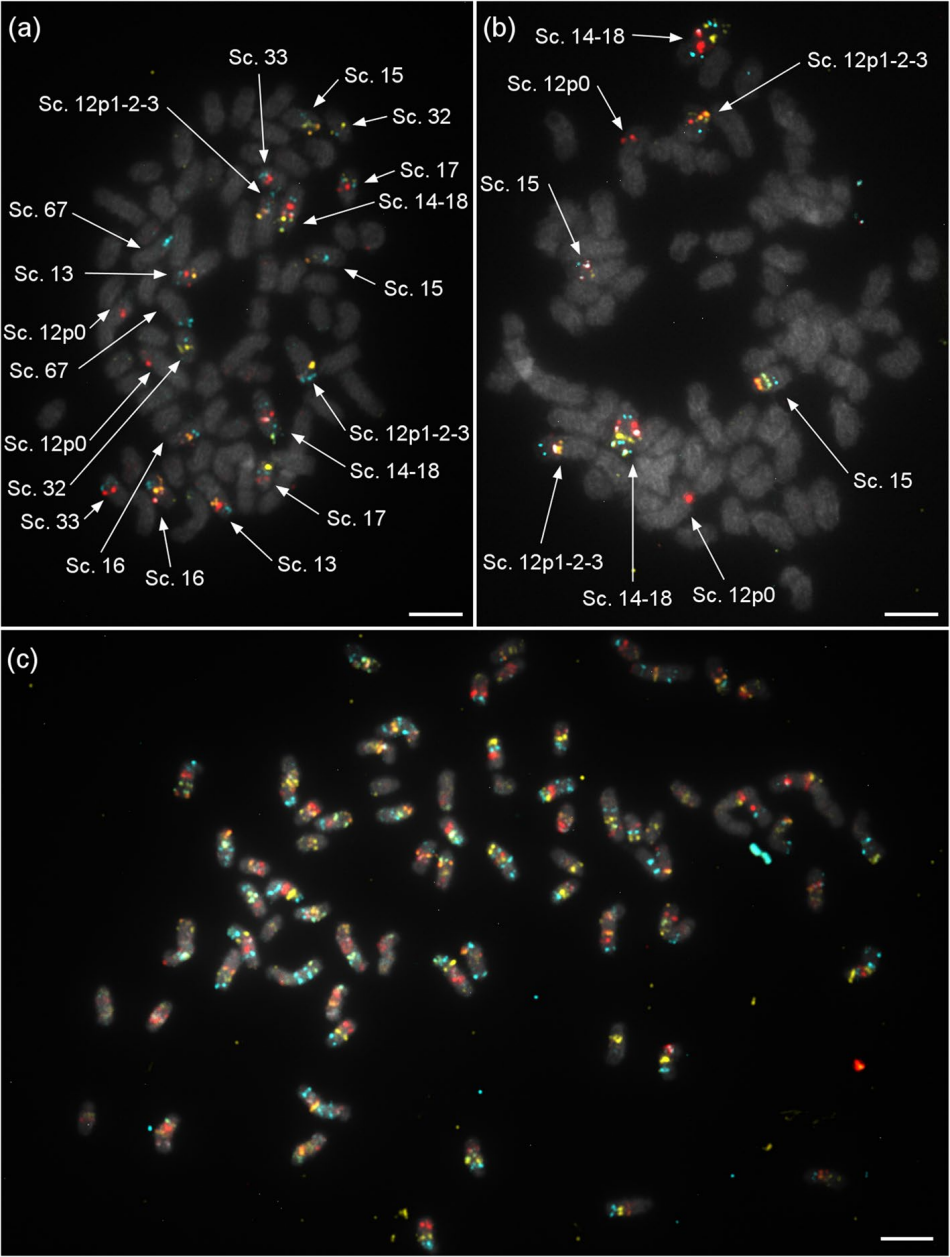
Hybridization of probes for scaffold mapping. **a** Hybridization of a group of ten scaffold probe sets, the first step for scaffold anchoring. Scaffold probes were labeled with FAM (cyan), ATTO-550 (yellow), ATTO-647 (red) or with a pair to generate another color (green for FAM + ATTO-550, orange for ATTO-550 + ATTO-647, violet for FAM + ATTO-647). **b** Example of unclear or mis-assembled signal scaffolds confirmed by hybridization in a smaller group. **c** Whole karyotype covered by the 68 scaffold probe sets allowing identification of all chromosomes with a single hybridization. All scaffolds were assigned to one pair of chromosomes by identifying their color scheme. Scale bar = 5 μm

The position of each of the 68 scaffolds was determined by hybridizing the corresponding probe sets alone or with the probe sets of other scaffolds to generate distinctive color patterns (Fig. 2). Initial hybridizations targeted presumptive chromosomal membership based on previous scaffold mapping of the bovine genome [20], which reduced total number of hybridization steps since it was not totally random. Full assembly was successfully achieved by permutating probe sets of a few scaffolds at a time for each chromosome spread hybridization and by comparing results across multiple hybridizations. Chromosomal mapping was confirmed by rehybridization of tandem scaffolds on additional chromosomal spreads. Scaffold mapping on both caribou and reindeer cells revealed no chromosomal differences between sub-species. Chromosome mapping of all 68 scaffolds was shown to cover all autosomes and a euchromatic part of the X chromosome (Fig. 2). These results showed that the oligo-based cytogenetic method can be successfully used for superscaffolding. Scaffolds were organized and oriented to significantly improve the reference genome quality. For instance, the resulting assembly is less fragmented as indicated by the significant increase in the N50 value (Table 1). At the 50% mark of the total genome assembly, the shortest contig is now of 54,365 kb and nearly doubled in size compared to the previous value. Larger genomic fragments also translate into a significantly lower L90 value decreasing by 37 scaffolds. This means that 90% of the entire genome (34 autosome pairs and the sex chromosomes) is now comprised into only 94 fragments (Table 1).

**Table 1:** Comparison of *R. tarandus* genome assemblies proposed by different authors

Publication	L90 number of scaffolds	N50 contig (kb)
(22)	–	986
(23)	289	11,765
(24)	–	5023
(20)	131	29,299
The present study	94	54,365

### Scaffold mis-assembly

**Fig. 3:**
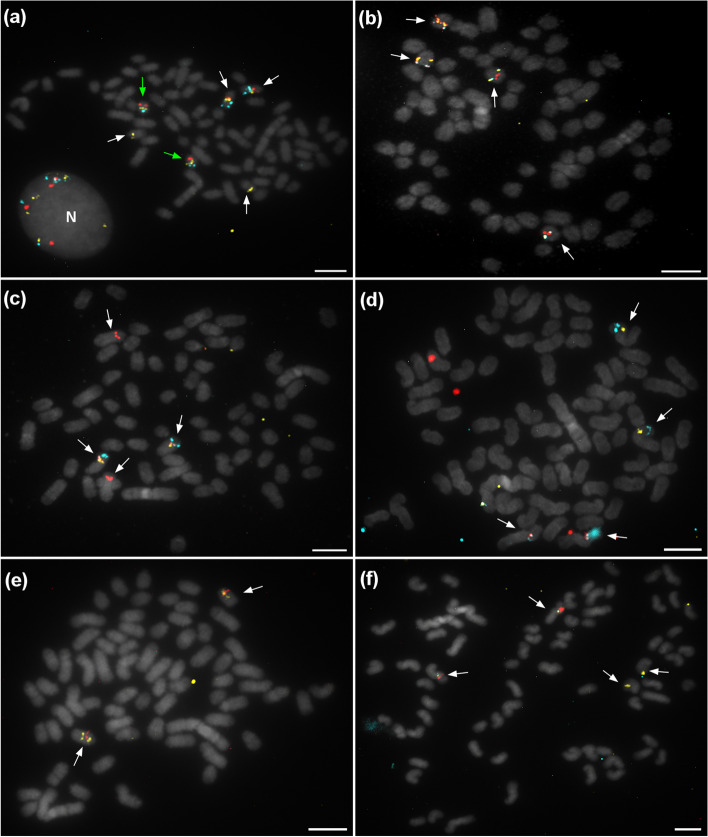
Validation of doubtful scaffolds integrity visualized on in situ chromosomes. White arrows in images **a**–**d** indicate mis-assembled scaffold splits on chromosome pairs: scaffold 1 on chromosome pairs 7 and 20, scaffold 2 on 16 and 18, scaffold 12 on 1 and 4, scaffold 20 on 2 and 34. Green arrows indicate well-assembled scaffold 7 on chromosome pair 27. Probes hybridized also with non-lysed cells (N). **e** Reorganized scaffold 21, the first part (including the first probe) is inverted and positioned after the second and third probes. **f** Mis-assembled scaffold 25 split on chromosome pairs 3 and 8. Scale bar, 5 μm

The putative location of each probe within scaffolds was validated by hybridization on several chromosomal spreads of both caribou and reindeer cells. Six of the 68 scaffolds initially assembled by bioinformatics were found to be mis-assembled. Five of these were in fact composed of sequences belonging to two different chromosomes (Fig. 3 and Fig. S1). Chimeric scaffolds were all confirmed by FISH block split on chromosomal spreads. Break points were determined based on synteny with the *Bos taurus* genome. These are presented in Table S1 along with ID of probes surrounding break points and the bovine chromosomal homologs to the scaffold blocks. The sixth mis-assembled scaffold (number 21) contained an ordering error, revealed by a colour pattern that was differently ordered than initially expected (Fig. 3e and Fig. S1e).

### Chromosome ordering and idiogram building

**Fig. 4:**
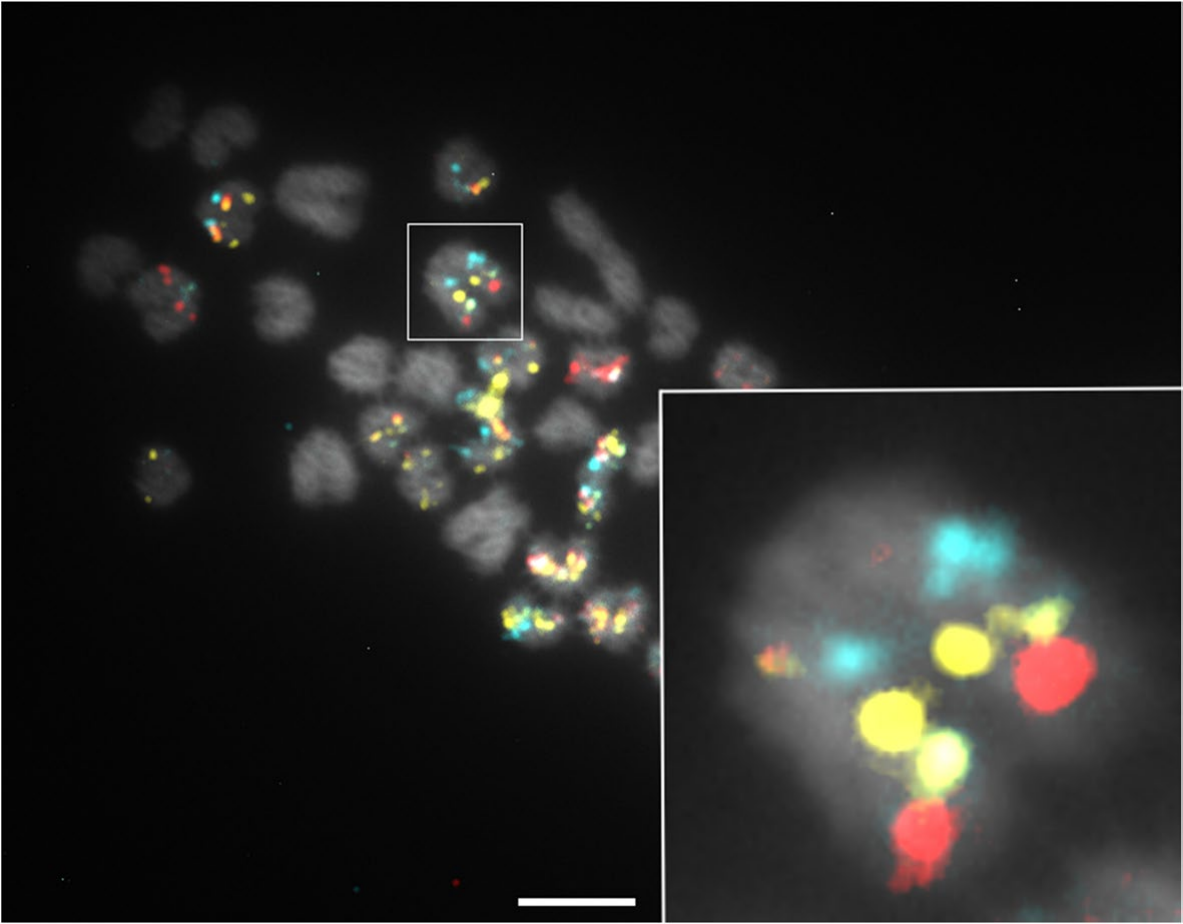
Centromere positioning revealed by hybridization on late metaphase chromosomes. More condensed chromosomes and clear separation of chromatids make centromere easier to distinguish. Inset: chromatid attachment point (centromere to the upper left side) visualized on *R. tarandus* chromosome 1. Scale bar = 5 μm

**Fig. 5:**
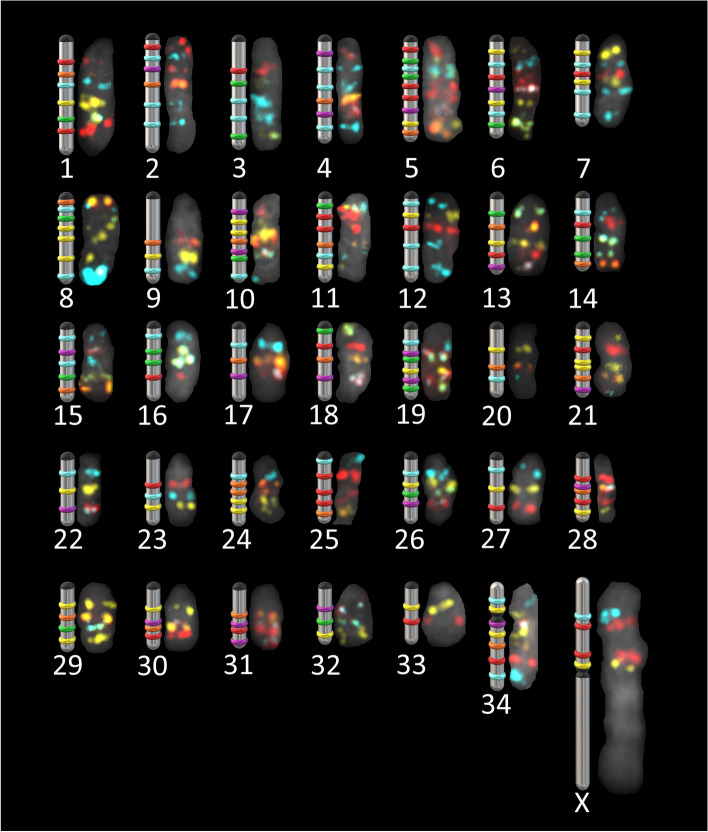
Idiographic and physical mapping of *Rangifer tarandus* scaffolds. All scaffolds were positioned on a chromosome. Centromeres are represented by black tips or bands. The first 33 chromosomes are acrocentric. The only submetacentric chromosome is #34. The X chromosome is the largest and is metacentric. All X chromosome scaffolds were placed on the short arm. The Y chromosome was not studied

Once all scaffolds were positioned, chromosome orientation was determined by positioning the centromeres. Given that nearly all autosomes are acrocentric and of similar size, centromere positions and scaffold orientation were determined by visualization of colour patterns on late metaphase chromosomes, since chromatids are easier to distinguish at this stage (Fig. 4). This method led to the positioning of all centromeres based on several observations for each chromosome. By cytogenetic convention, autosomes are usually grouped according to conformation or structure and ranked from the longest to the shortest. Here, chromosomes were ordered from acrocentric, submetacentric to metacentric and by decreasing length [30] (Fig. 5). Length was expressed relative to the X chromosome, which is the largest and therefore the most easily identifiable of all *Rangifer tarandus* chromosomes. Using this ratio avoided any length bias caused by chromosomal compaction related to cell mitotic cycle, which can vary across spreads. The relative lengths were used for chromosome ordering and numbering. Chromosome mapping of all 68 scaffolds and their respective lengths are presented in Table 2.

**Table 2:** *Rangifer tarandus* chromosome lengths and current scaffold mapping

Chromosome	Relative length (μm)	SD (μm)	Composition			
			Scaffold 1	Scaffold 2	Scaffold 3	Scaffold 4
1	6.126	0.542	43	8	12p0	–
2	6.075	0.415	49rev	20p2rev	5rev	–
3	5.926	0.319	25p1–2	4	–	–
4	5.623	0.480	6rev	12p1–2-3	–	–
5	5.473	0.296	42	65	37	55
6	5.266	0.279	68	14	18	–
7	4.728	0.284	48rev	1p0–1–1.1-1.2rev	–	–
8	4.700	0.121	66rev	22rev	25p0	67rev
9	4.651	0.352	9rev	–	–	–
10	4.587	0.263	24rev	62	34rev	–
11	4.567	0.331	30rev	19rev	–	–
12	4.565	0.538	63	3rev	–	–
13	4.245	0.556	45	27	–	–
14	4.149	0.300	11	60	–	–
15	4.132	0.193	36	15	–	–
16	4.130	0.378	50	2p0–1rev	–	–
17	4.100	0.493	16	–	–	–
18	4.078	0.321	57rev	2p2–3	–	–
19	4.026	0.341	52	54rev	61rev	–
20	3.945	0.048	1p2	35rev	–	–
21	3.867	0.068	21p1–2-(0rev)	29	–	–
22	3.815	0.212	10	–	–	–
23	3.791	0.325	17rev	–	–	–
24	3.777	0.434	51	23rev	–	–
25	3.731	0.257	33	31	–	–
26	3.726	0.348	32	46	–	–
27	3.691	0.164	7	–	–	–
28	3.614	0.187	59	26rev	–	–
29	3.576	0.345	40	39rev	–	–
30	3.516	0.220	41	58rev	–	–
31	3.394	0.224	47	44	–	–
32	3.220	0.371	28rev	–	–	–
33	3.217	0.151	38	–	–	–
34	5.538	0.160	20p0–1rev	56rev	13rev	–
X	10.892	0.561	64	53rev	–	–

*p* Scaffold probe number for mis-assembled scaffolds, *Rev* Reversed scaffold sequence according to centromere position

### Investigation of synteny and evolution

**Fig. 6:**
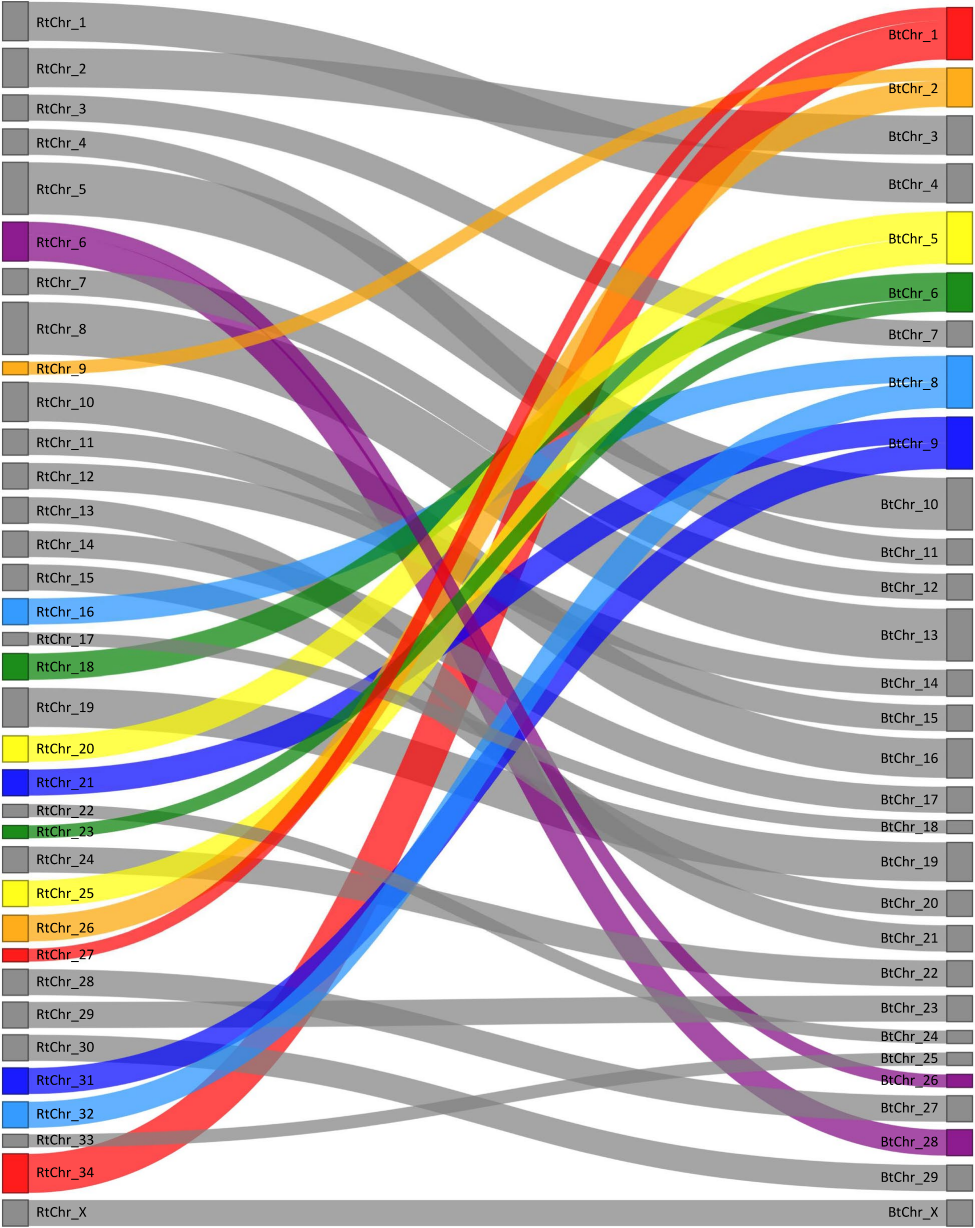
Sankey diagram illustrating associations between *Rangifer tarandus* chromosomes (RtChr, left) and *Bos taurus* chromosomes (BtChr, right). Chromosomal rearrangements (fusion and fissions) are shown in colour. Except for the coloured chromosomes, chromosomal order is maintained overall, the biggest discrepancy being of four chromosomal positions (i.e., RtChr13)

**Fig. 7:**
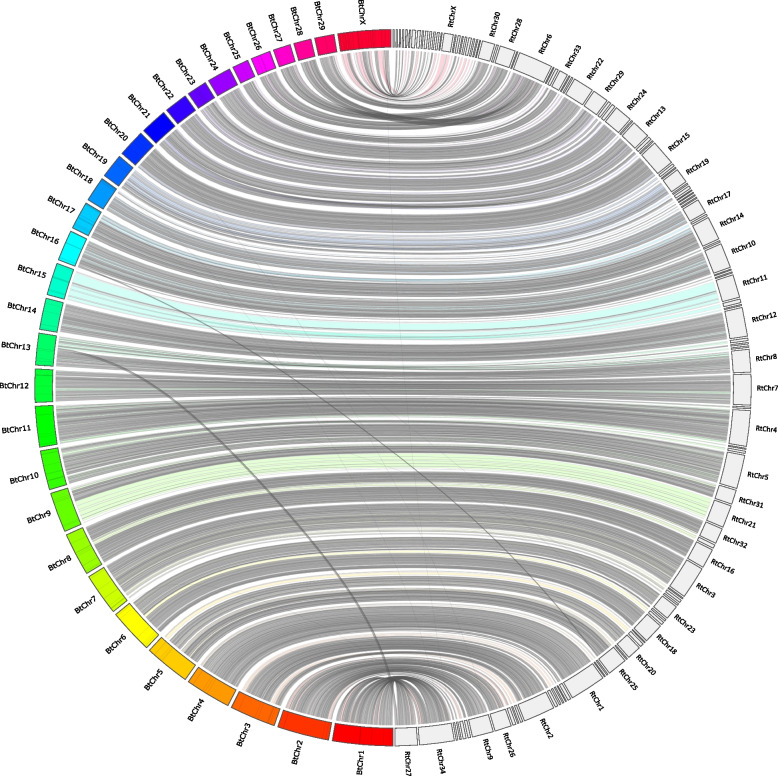
Jupiter plot representing mapping of the corrected *R. tarandus* assembly on the *B. taurus* genome. The 111 largest scaffolds (on the right) show high synteny with the 29 autosomes plus X chromosomes from the cattle assembly (ARS-UCD1.2). Intersecting bands, which represent non-syntenic regions between the two species, are fewer in comparison with the previous mapping [20]

**Fig. 8:**
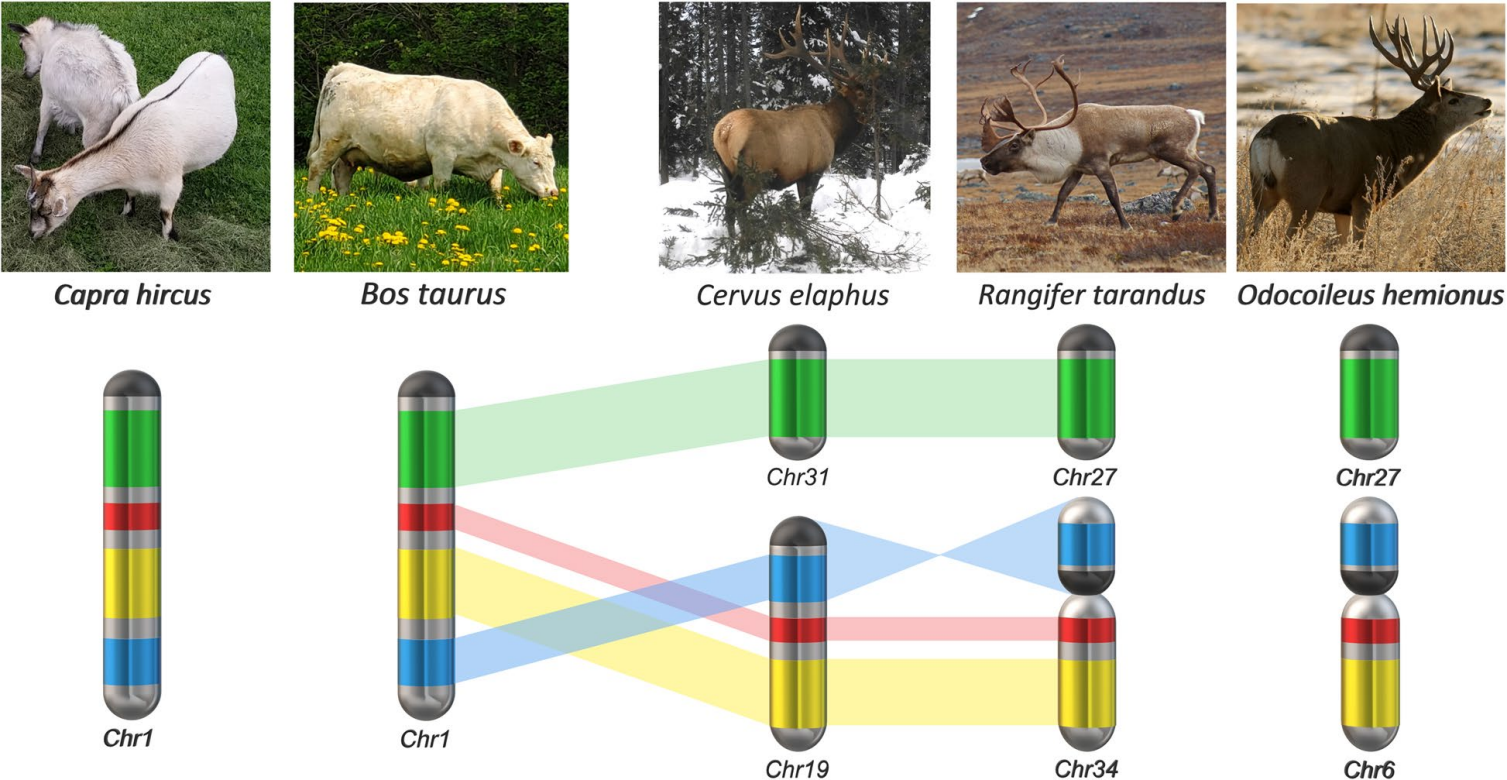
Suggested evolution of bovine chromosome 1 ancestral ortholog in Cervidae and Bovidae. Relative to bovine chromosome 1 and its caprine ortholog, three events appear to have been passed on to *R. tarandus* and *O. hemionus*. First, fission of the common ancestor’s bovine chromosome 1 ortholog gave rise to a short acrocentric chromosome (containing *R. tarandus* scaffold 7, green) and a longer one (containing scaffolds 13, 20 and 56, yellow, blue, red). A translocation then occurred within the distal portion of the longer one, near the centromere. The order of these two events has not been confirmed. Finally, a pericentric inversion of the proximal part of the longer chromosome occurred, leading to a submetacentric configuration in the genera *Odocoileus* and *Rangifer*

To test the value of this novel *Rangifer tarandus* reference genome assembly, we compared it first with bovine chromosomes. A previous bioinformatic comparison [20] predicted high synteny between the two species, which was confirmed by the observed chromosomal rearrangements and highlighted in Fig. 6. Correction of the six mis-assemblies that we found further emphasized synteny (Fig. 7). We then used the new assembly to model the evolution of chromosome 1 across related species by comparing orthologous sequences in species of Cervidae and Bovidae for which draft genomes are publicly available. Two chromosomal rearrangements were found common in all studied Cervidae species (*Cervus elaphus* (red deer), *R. tarandus* and *Odocoileus hemionus* (mule deer) compared to *Bos taurus* and *Capra hircus* (domestic goat) (Fig. 8). Additionally, a third rearrangement observed between *R. tarandus* and *Odocoileus hemionus* and the other species is suspected in their evolution (Fig. 8). The most parsimonious explanation for these rearrangements is that the common ancestral chromosome 1 first split in the Cervidae lineage to give rise to a small acrocentric chromosome (containing one *R. tarandus* scaffold, in green) and a larger chromosome (containing three scaffolds, in red, yellow and blue) (Fig. 8). A translocation within the larger resulting chromosome then relocated the distal portion near the centromere. The chronology of these two events were not determined though. Finally, a pericentric inversion of the proximal portion of the chromosome occurred, leading to a submetacentric configuration in the genera *Odocoileus* and *Rangifer* (Fig. 8).

## Discussion

The work presented herein leads to the development of a karyotyping method for *Rangifer tarandus*. Since most autosomes are similar in this species, the capacity to generate color banding patterns specific to each chromosomes provides an interesting tool for chromosome identification. Specific sets of probes can be selected and reamplified to highlight a subset of chromosomes or of chromosomal regions.

The color patterns allowed to significantly improve the genomic reference built from scaffolds assembled bioinformatically to a chromosome-level assembly. Completely sequenced chromosomes represent major scientific advances which undoubtedly represent a valuable resource for further genomic studies. However, very seldom are initial draft genomes assembled to this extent. The use of proximal tagging strategies and various DNA sequencing platforms that provide different coverage and read lengths can yield sufficient data to generate super-scaffolds [2, 3, 5, 46], but even these large genomic fragments rarely cover entire chromosomes. One of the main obstacles is the presence of repeated elements that make it impossible to establish with certainty the relative positions of scaffolds [5]. Cytogenetics offers a complementary strategy to bioinformatics by physically positioning sequences of interest through in situ hybridization [4, 7, 44, 47–51].

To date, animals for which complete chromosome-mapped genomes are available for reference purposes include humans [52], mice [53], *Drosophila* [54], zebra fish [55], chickens [56], cattle [57], swine [58], sheep [59], and goats [60]. However, several wild species of conservation concern have not been fully chromosome-assembled [3, 4, 6, 61]. For instance, four *Rangifer tarandus* draft genome assemblies have been published in recent years [20, 22–24]. However, none have reached chromosome-level assembly. Herein, we used Oligopaint FISH probes (modifiable synthetic short oligonucleotides) to anchor the 68 currently largest *Rangifer tarandus* scaffolds from the most recent *R. tarandus* genome [20] to chromosomes. The oligonucleotide-based technology was chosen instead of the de novo synthesis of DNA probes from bacterial artificial chromosomes (BAC) since this latter method is less efficient and more expensive [7, 62–64]. Another alternative could have been to use existing probe-sets developed in other species but even with meticulous sequence selection, hybridization success rate can vary considerably [48]. Moreover, these probes generally target both single-copy sequences and repetitive sequences, which significantly reduces specificity and possible applications [65].

Long synthetic oligo have been used to probe genomic regions as small as 6.7 kb [33, 35]. Shorter probes (≤ 100 bp) have been effective for 10 kb regions [34, 36], albeit more efficient when targeting 52 kb to 2.1 Mb to hybridize on nuclei [36]. The probe sets designed in our study targeted 401.5 kb on average, a length for which our probe sets containing 1500 oligo appeared to be optimal. Mean oligo density per probe was thus 3.74/kb, lower than the 5.5/kb previously used for targeting 500 kb [36]. However, mean densities as low as 1.71/kb have been used successfully to hybridize with 500 kb sequences chosen within chromosomal sets [42]. Our hybridization results show that small variations in the number of oligo per probe, oligo density and the targeted sequence length has little impact on detection, and that the method is therefore robust and flexible.

Another parameter that could influence probe detection in oligo-based technology, particularly the resolution at which probes are distinguishable from each other, is inter-probe distance. In the present study, the lowest inter-probe distance (excluding the distance between probes of adjacent scaffolds) was 7.4 Mb, that is, between the first and second probes of chromosome 8. This is consistent with previous studies in which a 7–8 Mb gap between probes was sufficient [33, 41]. The minimal inter-probe distance to ensure acceptable probability of detection and visualization is the main reason why smaller scaffolds could not be used. Overall, our probe design parameters allowed us to map all selected scaffolds, which account for 78% (2.01 Gb) of the entire *R. tarandus* genome. This coverage is comparable to other bioinformatic and FISH-assisted genome assemblies [4, 48].

We are also proposing a new *R. tarandus* idiogram based on fluorescent banding patterns. Previous published karyotypes differently ordered chromosomes, and banding patterns were most often of low resolution to clearly identify *R. tarandus* chromosomes especially since they are nearly all acrocentric and many are of similar size [30, 66, 67]. Moreover, the submetacentric chromosome was differently placed across karyotypes, either placed as the first autosome [67], or the last one [25, 30, 31] or amongst the acrocentric [66]. Herein, ordering is based on chromosome size evaluated by repeated length measurements and placing the submetacentric chromosomes as the last autosomes. A recent publication reports a higher resolution *R. tarandus* G-banding karyotype offering a different chromosomal ordering where the submetacentric pair is not identified [68]. While we used *B. taurus* as reference for comparative analysis, Proskuryakova and colleagues used a comparative approach using probes derived from *Camelus dromedarius* [68]. The two reference species are known to harbour several evolutionary chromosomal rearrangements [69] preventing clear chromosome identification between studies.

While the probe-sets were designed to position and orient all selected scaffolds onto the chromosomal spreads, since the initial scaffolds were obtained from a bioinformatics assembly, the designed probe-sets were also confirming the existence of the in silico derived fragments and allowed the detection of chimeric sequences. A total of 18 breaks in synteny were previously identified when mapping *R. tarandus* scaffolds to a bovine genome [20]. Such analysis cannot distinguish true chromosomal rearrangements from chimeric assembly. The split scaffolds identified in the present study match five of the seven largest previously identified potential inter-species discrepancies, confirming that these were examples of scaffolding errors. Furthermore, colour patterns did reveal an intra-scaffold rearrangement. All corrected scaffolds were among the 40% largest, which seems concordant with the increased number of sequence matching events needed to lengthen the scaffolds. Several reference genomes have been corrected after publication by visualizing potential errors through FISH probe hybridization [6–8], thus supporting the usefulness of cytogenetics in genome assembly. Corrected scaffolds reduced syntenic breaks observed previously [20] and further confirmed the reported high synteny between Cervidae and Bovidae [22, 23, 70].

Despite this high synteny between Cervidae and Bovidae, chromosomal rearrangements that were not highlighted in the previous genome mapping [20] were revealed by cytogenetics. Several studies comparing species in the infraorder Pecora revealed evolving chromosomal rearrangements [25, 47, 49, 71–75]. A chromosome painting study comparing *B. taurus* and several deer species including red deer, milu deer (*Elaphurus davidianus*), rusa deer (*Cervus timorensis russa*), Eld’s deer (*Rucervus eldii*), fallow deer (*Dama dama*), roe deer (*Capreolus capreolus*), Chinese muntjac (*Muntiacus reevesi*) and moose (*Alces alces*) has revealed karyotype differences traceable to fission of cattle chromosomes 1, 2, 5, 6, 8 and 9 and tandem fusion of cattle chromosomes 26 and 28 [49]. Our results support the same karyotypic evolution and suggest that bovine chromosome 28 has the same centromeric region as *R. tarandus* chromosome 6 (Fig. S2). We therefore hypothesize that bovine chromosome 26 centromere formed after the fission. The centromere of bovine chromosome 28 has been associated also with the centromere of *C. elaphus* chromosome 15, which also contains both bovine chromosomes 26 and 28 [70]. Although fission of *B. taurus* chromosomes 26 and 28 was unambiguously predicted by bioinformatics [20], fission of chromosomes 1, 2, 5, 6, 8 and 9 was not, thus showing the usefulness of physical mapping.

Bovine chromosome 1 represents an interesting case as it has been associated with many chromosomal rearrangements among Cetartiodactyla [49]. In nine Cervidae species studied in that review, bovine chromosome 1 was found to be split into a smaller acrocentric chromosome and a larger acrocentric or submetacentric chromosome. In our mapping, the scaffold associated with the proximal part of bovine chromosome 1 is located alone on the small acrocentric *R. tarandus* chromosome 27, and the distal part is located on the submetacentric *R. tarandus* chromosome numbered 34.

To explore Cervidae chromosomal evolution further, we mapped the bovine first chromosome related scaffolds to the latest versions of the mule deer genome (*Odocoileus hemionus;* GCA_020976825.1) and the red deer (*Cervus elaphus;* GCA_910594005.1) genome. It has been reported that the distal portion of the larger chromosome resulting from the split of the bovine chromosome has undergone a translocation to the middle/proximal region in several Cervidae species [47, 49, 70]. Furthermore, in the Capreolinae subfamily, containing the genera *Odocoileus*, *Rangifer* and *Alces* among others, a pericentric inversion within a large acrocentric chromosome leading to a submetacentric type has been reported [25, 29, 71]. Based on suggested karyotype evolution [25, 71], cross-species hybridization [49] and genome assembly [70], *C. elaphus* does not contain this pericentric inversion. Our mapping shows the same scaffold order on *C. elaphus*, *O. hemionus* and *R. tarandus*, suggesting that these three cervid genomes contain the same translocation. The pericentric inversion was not confirmed directly by FISH since no non-inverted chromosome was probed for comparison. However, BAC probe hybridization results for *R. tarandus* chromosomes [49] and our *R. tarandus* assembled genome comparison with *B. taurus* genome (Fig. S3) tend to support this rearrangement. Based on these observations, we suggest a karyotype evolution scheme including *Bos taurus*, *Capra hircus*, *Cervus elaphus*, *Rangifer tarandus* and *Odocoileus hemionus* (Fig. 8) in which fission of an ancestral bovine chromosome 1 ortholog gave rise to a small acrocentric *R. tarandus* chromosome containing one scaffold (in green) and a larger one containing three scaffolds (yellow, blue, and red) within which a translocation moved the distal portion to near the centromere in extant Cervidae. The chronology of these two chromosomal rearrangement events remains to be determined. Finally, a pericentric inversion of the proximal portion occurred, leading to a submetacentric configuration in the genera *Odocoileus* and *Rangifer* (Fig. 8). We expect that both the translocation and the pericentric inversion occurred in *Alces alces* and other *Odocoileus* species since their karyotypes are closely related according to previous phylogenetic mapping [25]. Further FISH experiments will be needed to test this hypothesis. Since cross-species hybridization can sometimes proves to be informative specially to confirm specific evolutionary genomic reorganizations [47, 49], all probes developed herein for *R. tarandus* have been made available (supplemental data).

## Conclusions

Mapping 78% of the *Rangifer tarandus* genome onto chromosomes adds considerable value to the reference genotyping of this species. These results will provide resources for future studies of caribou and reindeer phylogenetics, conservation genetics and cytogenetics. The Cervidae family is remarkable for the high diversity of its chromosome shapes and numbers, which represents both challenges and opportunities for scientific research. *Rangifer* comprises several species and subspecies that have yet to be fully characterized genetically. Since many circumpolar caribou and reindeer populations are listed as endangered or threatened, precise genomic information for conservation and management will become an important asset.

## Methods

All chemicals were purchased from ThermoFisher (Mississauga, ON, Canada) unless specified otherwise.

### Probe design

Probes were designed as described previously [45] using 1500 synthetic oligo by probe. Each oligo consisted of a scaffold-specific 39-mers homolog flanked by reverse and forward primer sequences, the latter extended by a 5′ adapter complementary to a fluorophore-bearing detector sequence, as shown in Fig. 1. The primers, adapter and detector sequence were all 20-mers. The 39-mers sequences were designed on the *Rangifer tarandus* repeat masked genome [20] using OligoMiner tools [38] in the “balance” default mining mode with few exceptions. The minimal and maximal lengths were set at 39-mers in *blockParse.py (−l and -L)* to ensure candidate length homogeneity. *Unique Mode* (UM) and *Linear Discriminant Analysis* Mode (LDM) were used for Bowtie2 alignment and outputClean.py steps. Results from the previous step were compared and oligo in common were chosen. Candidates were filtered by running the optional *kmerFilter.py* script then *structureCheck.py* with a simulated hybridization temperature (*−T*) set at 42 °C. This eliminated high-abundance k-mers and secondary structures among candidate probes.

*Ifpd* tools v2.0.4 [45] were then used to design and select probes. Default parameters were used except for ifpd_query_set, which was run with *--order centrality homogeneity size --n-oligo 1500.* Probe sets were chosen based on distance between probes and from ends of scaffolds to ensure sufficient inter-probe distance for microscopic resolution. The number of probes per scaffold was set according to scaffold length and the maximal number that filled three 92 K Genscript oligo libraries. Selected 39-mers are shown in Additional file 1.

Orthogonal primer sequences were generated as described previously [45]. Briefly, the 240,000 orthogonal 25-mers designed previously [76] were each sliced into six 20-mers to generate a total of 1,440,000 20-mers. The *OOD-FISH* pipeline [45] was then used with default parameters but with *BLAST* v2.7 to align the sequences to the non-repeat masked *Rangifer tarandus* genome [20] with the following parameters: *blastn -word_size 6 -evalue 1000 -penalty − 2 -reward 1 -task ‘blastn’ -outfmt 6* instead of BLAT. Sequences with an e-value < 25 were filtered out to isolate candidates with high-quality homology with the genome. Retained sequences were then filtered for self and hetero-dimers (SDFE and HDFE filters, respectively) and sequences with a free energy ≥ − 9 kcal/mol were kept. Candidates with 5′ GC clamp were preferably attributed to reverse primers and ones with clamp on 3′ to forward primers to promote stronger binding during amplification and transcription, others were distributed randomly. To preserve fluorophore interchangeability, forward primer and adapter sequences were fluorophore specific. Reverse primer sequences were scaffold-specific drawn from a library for individual amplification purposes. All orthogonal sequences used are listed in Additional file 2.

Genomic 39-mers homologous and primer sequences were assembled in final 79-mers and were purchased from *GenScript Biotech* (Piscataway, NJ, USA). They are listed in Additional file 1. Primers and detection oligo were purchased as standard desalted and HPLC purified oligo respectively from Integrated DNA technologies (IDT) and are listed in Additional file 3.

### Colour attribution

Colour schemes for scaffold detection and identification were assigned avoiding scheme repetitions within a library to ensure scaffold identification within a hybridization pool. Fluorophores used for each probe are listed in Additional file 4. Colours were generated using 6-FAM, ATTO 550, or ATTO 647 N bound to the 3′ end of the fluorophore-bearing oligo (Additional file 3). To obtain additional colours (green, orange, or violet), probes were made with two adapters specific for different fluorophores.

### Probe synthesis

Probes were produced as described previously [77] with slight modifications. Briefly, oligo libraries were amplified by real-time PCR using PerfeCTa SYBR Green Fastmix (Quantabio, Beverly, MA, USA) in a LightCycler 480 II (Roche, Rotkreuz, Switzerland) to analyze amplification curves. Reagents were added as described elsewhere [77]. The T7 RNA polymerase recognition site was added with reverse primers (5′ end) for in vitro transcription. Probes were generated individually in separate wells by adding the corresponding oligo library and the specific reverse primer for each scaffold. Forward primers with specific 5′ detection adapter sequences were added to the reaction mixture. The PCR product was purified using SparQ PureMag Beads (Quantabio, Beverly, MA, USA) and quantified using a NanoDrop One (ThermoFisher Scientific) according to standard (manufacturer’s) instructions.

Purified PCR products were transcribed in vitro using a HiScribe T7 High Yield RNA Synthesis Kit (New England BioLabs, Ipswich, MA, USA) according to the manufacturer’s instructions but with 1 μL of RNAseOut Recombinant Ribonuclease Inhibitor per 20 μL total reaction volume. The reaction time was set to 12–16 h in a C1000 Touch Thermal Cycler (Bio-Rad, Mississauga, ON, Canada). DNA was removed by digestion with DNase I (New England BioLabs) and RNA was purified with RNA Clean XP (Beckman Coulter, Mississauga, ON, Canada) and quantified using a NanoDrop One (ThermoFisher Scientific) according to the manufacturer’s instructions.

Purified RNA was reverse-transcribed using Maxima H Minus Reverse Transcriptase as described previously [77] with 5 μg of template RNA in 20 μL total reaction volume. Complementary DNA was purified using Zymo-Spin IC Columns (Cedarlane, Burlington, ON, Canada) and quantified using a NanoDrop One (ThermoFisher Scientific, ON, Canada) according to the manufacturer’s instructions.

### Cell culture and sample preparation

Cryopreserved fibroblast cells available at the Toronto Zoo were used for karyotyping. Cells were originally derived from biopsy punches of a female caribou (Porcupine herd, Canada, *Rangifer tarandus granti*) and a female Eurasian tundra reindeer (*Rangifer tarandus tarandus*) both housed at the Toronto Zoo (Ontario, Canada). Cells were thawed and cultured at 37 °C in T-25 flasks containing Multicell DMEM/F12 medium (cat. 319–085-CL, Wisent Inc., St-Bruno, QC, Canada) supplemented with 20% fetal bovine serum (Wisent Inc) and 1% Penicillin-Streptomycin (Wisent Inc) in a humidified 5% CO_2_ atmosphere. Cells were harvested as described in a published protocol [78]. Briefly, culture at 80% confluence was treated with KaryoMAX™ Colcemid™ solution (cat. #15210040) for 45 minutes followed by hypotonic potassium chloride (0.075 mol/L) and Carnoy’s fixative steps. Fixed cells were dropped from a height of 15 cm onto microscope slides in a 50% humidity and room temperature atmosphere. Slides were aged for 24 h at room temperature before hybridization.

### Hybridization of Oligopaint FISH probes

Probes were hybridized following a protocol described elsewhere [38] with some modifications. Briefly, slides were immersed first in 70 °C 70% formamide in 2X saline-sodium citrate buffer (SSC) for 2 min then in 70, 90 and 100% ethanol at − 20 °C for 3 min each. The 79-mers hybridization mix, containing 0.4 μmol/L of each scaffold probe in 50% formamide and 10% dextran sulfate in 2X SSC, was added (40 μL) to each slide, covered with a LifterSlip (Electron Microscopy Sciences, Hatfield, PA, United States) and hybridized for 16–18 h at 40 °C in Array Booster AB410 (Advalytix AG, Brunnthal, Germany) humidified chambers. Slides were immersed in 0.1% Tween 20 in 2X SSC at 60 °C to remove the LifterSlip, soaked for 15 min, transferred twice to fresh Tween 20 buffer at ambient temperature for 5 min each then air-dried.

For hybridization of detection oligo, 40 μL of hybridization II mix (3 μmol/L of each labelled detection oligo in 30% formamide in 2X SSC) was placed on a slide, covered with a LifterSlip and hybridized at ambient temperature for 1 h. The LifterSlip was removed using the Tween 20 buffer at ambient temperature and the slide was washed twice for 10 and 2 min (same buffer) then in 0.2X SSC for 2 min. Excess buffer was drained and True View autofluorescence quenching was carried out according to the manufacturer’s instructions (Vector Laboratories, Burlingame, CA, United States). Samples were mounted in Vectashield Vibrance antifade medium with DAPI (Vector Laboratories) and cured for at least 2 h before imaging.

### Imaging and analysis

Imaging was performed on a Nikon Eclipse e600 microscope with a Nikon C-SHG1 Super High-Pressure Mercury Lamp and a 100x oil immersion Nikon objective. Filters were obtained from Nikon or Chroma, and the following excitation/emission wavelength ranges (in nm) were used: 340–380/435–485 for DAPI, 465–495/515–555 for 6-FAM, 510–560/> 570 for ATTO 550, and 625–655/665–715 for ATTO 647 N. Images were acquired using a QImaging EXi Blue CCD Camera and QCapture Pro 7 software. Image production and analysis were achieved using Fiji ImageJ [79]. Paint 3D was used for Figs. 2 and 4. Straightening tools from ImageJ were used for karyotype images of highly curved chromosomes (Fig. 2). Figure 3 was produced using the *sankeyNetwork* function in *networkD3* package v0.2.4 [80] on R. Synteny with bovid and other cervid genomes was investigated using minimap2 [81] and visualized using the JupiterPlots bioinformatic tool on Linux [82].

## Supplementary Information

**Additional file 1.** Information on 79-mers used for chromosome-level assembly. These Excel files list each oligo with its unique ID and associated probe on a scaffold. The 39-mers genome homologous sequences obtained from running OligoMiner and IFPD scripts are also given with their start and end positions on the scaffold sequence. R_id and F_id are based on 20-mers ID attribution (Additional file 2). The reverse and forward specific 20-mers sequences were assigned according to scaffold number and colour pattern respectively. Each 20-mers forward sequence bears a unique fluorophore (Fluorophore column). Assembled 79-mers oligo sequences were purchased from Genscript.

**Additional file 2.** Orthogonal 20-mers sequences and attribution. Excel file listing the 29 orthogonal 20-mers sequences by ID and used as a reverse or forward primer or a detection oligo. Reverse sequences were used on up to four scaffolds but were unique to a single scaffold in each library. Forward sequences and adapters were used for all libraries.

**Additional file 3.** Primers and detection oligo. Excel file listing the primers used for amplification and transcription reactions and as detection oligo. Reverse primers consist of the 20-mers complementary sequence linked to the 3’ end of the T7 sequence 5’-CGATTGAGGCCGGTAATACGACTCACTATAGGG-3’ [45]. Forward primer 20-mers are linked to the 3’ end of the adapter sequence. The resulting 53-mers reverse primer and 40-mers forward primer oligo were used for PCR. Forward primers were used also for RT-PCR. The fluorophore-linked homolog of the specific 20-mers adapter was the detector of probe binding. These 30 oligo were purchased from IDT as custom standard or HPLC-purified DNA.

**Additional file 4.** Scaffold probe fluorophore associations. Excel file listing the fluorophores used for each scaffold, based on scaffold colour scheme. To create additional colours, some probes were paired with two fluorophores to obtain a mixture.

**Additional file 5: Fig. S1.** *Rangifer tarandus* scaffold mis-assembly revealed by comparison with *Bos taurus* chromosomes. (a) Scaffold 1 mapped to bovine chromosomes 12 and 5; (b) Scaffold 2 mapped to bovine chromosomes 8 and 6; (c) Scaffold 12 mapped to bovine chromosomes 11 and 4; (d) Scaffold 20 mapped to bovine chromosomes 3 and 1; (e) Rearranged scaffold 21 mapped to bovine chromosome 9; (f) Scaffold 25 mapped to bovine chromosomes 13 and 7. Exact breakpoints are listed in Table S1. Green rectangles represent centromere positions.

**Additional file 6: Fig. S2.** Scaffolds 18, 14 and 68 (top to bottom order) forming *R. tarandus* chromosome 6 and corresponding to split bovine chromosomes 26 and 28. The fission that led to the new chromosomes has been carried in scaffold 14. The centromere of *R. tarandus* chromosome 6 apparently has been conserved in bovine chromosome 28 based on genome mapping. Green rectangles represent centromere positions.

**Additional file 7: Fig. S3.** Mapping of chromosomes 27 and 34 as assembled in this study on bovine chromosome 1 . Chromosomal fission led to the formation of the two *R. tarandus* chromosomes. The distal portion of the larger chromosome subsequently underwent translocation and inversion, the latter rearrangement creating the submetacentric character of *R. tarandus* chromosome 34. Green rectangles represent centromere positions.

**Additional file 8: Table S1.** Breakage positions in the mis-assembled scaffolds.

## Data Availability

The datasets supporting the conclusions of this article are included within the article (and its additional files). The chromosome-level genome version is available in the NCBI genome database: accession number JAHWTM000000000, https://www.ncbi.nlm.nih.gov/data-hub/genome/GCA_019903745.1/.

## Abbreviations

BAC: Bacterial artificial chromosome
BtChr: *Bos taurus* chromosome
FISH: Fluorescence in situ hybridization
Oligo: Oligonucleotide(s)
RtChr: *Rangifer tarandus* chromosome
SSC: Saline-sodium citrate buffer

## References

[1] Kim J, Larkin DM, Cai Q, Asan ZY, Ge RL (2013). Reference-assisted chromosome assembly. Proc Natl Acad Sci.

[2] Rhie A, McCarthy SA, Fedrigo O, Damas J, Formenti G, Koren S (2021). Towards complete and error-free genome assemblies of all vertebrate species. Nature.

[3] Fierst JL (2015). Using linkage maps to correct and scaffold de novo genome assemblies: methods, challenges, and computational tools. Front Genet.

[4] Waterhouse RM, Aganezov S, Anselmetti Y, Lee J, Ruzzante L, Reijnders MJMF (2020). Evolutionary superscaffolding and chromosome anchoring to improve Anopheles genome assemblies. BMC Biol.

[5] Luo J, Wei Y, Lyu M, Wu Z, Liu X, Luo H (2021). A comprehensive review of scaffolding methods in genome assembly. Brief Bioinform.

[6] O’Connor RE, Farré M, Joseph S, Damas J, Kiazim L, Jennings R (2018). Chromosome-level assembly reveals extensive rearrangement in saker falcon and budgerigar, but not ostrich, genomes. Genome Biol.

[7] Chamala S, Chanderbali AS, Der JP, Lan T, Walts B, Albert VA (2013). Assembly and validation of the genome of the nonmodel basal angiosperm Amborella. Science.

[8] Shearer LA, Anderson LK, de Jong H, Smit S, Goicoechea JL, Roe BA (2014). Fluorescence in situ hybridization and optical mapping to correct scaffold arrangement in the tomato genome. Genes Genome Genet.

[9] Wilson DE, Reeder DM (2005). Mammal species of the world: a taxonomic and geographic reference.

[10] Vors LS, Boyce MS (2009). Global declines of caribou and reindeer. Glob Chang Biol.

[11] Festa-Bianchet M, Ray JC, Boutin S, Côté SD, Gunn A (2011). Conservation of caribou (Rangifer tarandus) in Canada: an uncertain future. Can J Zool.

[12] Harding LE (2022). Available names for Rangifer (Mammalia, Artiodactyla, Cervidae) species and subspecies. ZooKeys.

[13] Cronin M, Macneil M, Patton J (2005). Variation in mitochondrial DNA and microsatellite DNA in caribou (Rangifer tarandus) in North America. J Mammal.

[14] Government of Canada (2021). Caribou in Canada.

[15] Mallory CD, Boyce MS (2018). Observed and predicted effects of climate change on Arctic caribou and reindeer. Environ Rev.

[16] Riseth JÅ, Tømmervik H, Forbes BC (2018). Sustainable and resilient reindeer herding. Reindeer and Caribou – health and diseases.

[17] Wittmer HU, McLellan BN, Seip DR, Young JA, Kinley TA, Watts GS (2005). Population dynamics of the endangered mountain ecotype of woodland caribou ( *Rangifer tarandus caribou* ) in British Columbia, Canada. Can J Zool.

[18] Uboni A, Horstkotte T, Kaarlejärvi E, Sévêque A, Stammler F, Olofsson J (2016). Long-term trends and role of climate in the population dynamics of eurasian reindeer. PLoS One.

[19] MFFP. Revue de littérature sur les facteurs impliqués dans le déclin des populations de caribous forestiers au Québec et de caribous montagnards de la Gaspésie. 2021. https://consultation.quebec.ca/uploads/decidim/attachment/file/122/RevueLitterature_CaribouVF.pdf. Accessed 13 Sept 2022.

[20] Prunier J, Carrier A, Gilbert I, Poisson W, Albert V, Taillon J (2021). CNVs with adaptive potential in Rangifer tarandus: genome architecture and new annotated assembly. Life Sci Alliance.

[21] Carrier A, Prunier J, Poisson W, Trottier-Lavoie M, Gilbert I, Ferchaud AL, et al. Design and validation of a 63K genome-wide SNP- genotyping platform for caribou/reindeer (Rangifer tarandus). BMC Genomics. In press. 10.1186/s12864-022-08899-6 PMC9533608 36199020

[22] Li Z, Lin Z, Ba H, Chen L, Yang Y, Wang K (2017). Draft genome of the reindeer (Rangifer tarandus). GigaScience.

[23] Taylor RS, Horn RL, Zhang X, Golding GB, Manseau M, Wilson PJ (2019). The caribou (Rangifer tarandus) genome. Genes.

[24] Weldenegodguad M, Pokharel K, Ming Y, Honkatukia M, Peippo J, Reilas T (2020). Genome sequence and comparative analysis of reindeer (Rangifer tarandus) in northern Eurasia. Sci Rep.

[25] Fontana F, Rubini M (1990). Chromosomal evolution in cervidae. Biosystems.

[26] Lee C, Ritchie DBC, Lin CC (1996). A tandemly repetitive, centromeric DNA sequence from the Canadian woodland caribou (Rangifer tarandus caribou): its conservation and evolution in several deer species. Chromosom Res.

[27] Nes N, Amrud J, Tøndevold OB (1965). Kromosomstudier hos rein (Rangifer tarandus). Nordisk veterinaermedicin.

[28] Fraccaro M, Gustavsson I, Hultén M, Lindsten J, Tiepolo L (1968). Chronology of DNA replication in the sex chromosomes of the reindeer (Rangifer tarandus L.). Cytogenetic Genome Res.

[29] Gripenberg U, Nieminen M (1986). The chromosomes of reindeer (Rangifer tarandus). Rangifer.

[30] Gripenberg U, Huuhtanen S, Wessman M, Nieminen M (1991). A fragile site in the X chromosome of the reindeer (Rangifer tarandus L). Genet Sel Evol.

[31] Dirsch O, Ji Y, Bohr J, Shen K, Levison D, Dahmen U (2007). Probe production for in situ hybridization by PCR and subsequent covalent labeling with fluorescent dyes. Appl Immunohistochem Mol Morphol.

[32] Raudsepp T, Chowdhary BP, Murphy WJ (2008). FISH for mapping single copy genes. Phylogenomics.

[33] Bienko M, Crosetto N, Teytelman L, Klemm S, Itzkovitz S, van Oudenaarden A (2013). A versatile genome-scale PCR-based pipeline for high-definition DNA FISH. Nat Methods.

[34] Boyle S, Rodesch MJ, Halvensleben HA, Jeddeloh JA, Bickmore WA (2011). Fluorescence in situ hybridization with high-complexity repeat-free oligonucleotide probes generated by massively parallel synthesis. Chromosom Res.

[35] Yamada NA, Rector LS, Tsang P, Carr E, Scheffer A, Sederberg MC (2011). Visualization of fine-scale genomic structure by oligonucleotide-based high-resolution FISH. Cytogenetic Genome Res.

[36] Beliveau BJ, Joyce EF, Apostolopoulos N, Yilmaz F, Fonseka CY, McCole RB (2012). Versatile design and synthesis platform for visualizing genomes with Oligopaint FISH probes. Proc Natl Acad Sci.

[37] Han Y, Zhang T, Thammapichai P, Weng Y, Jiang J (2015). Chromosome-specific painting in cucumis species using bulked oligonucleotides. Genetics.

[38] Beliveau BJ, Kishi JY, Nir G, Sasaki HM, Saka SK, Nguyen SC (2018). OligoMiner provides a rapid, flexible environment for the design of genome-scale oligonucleotide in situ hybridization probes. Proc Natl Acad Sci.

[39] Rosin LF, Gil J, Drinnenberg IA, Lei EP (2021). Oligopaint DNA FISH reveals telomere-based meiotic pairing dynamics in the silkworm, Bombyx mori. PLoS Genet.

[40] Qu M, Li K, Han Y, Chen L, Li Z, Han Y (2017). Integrated karyotyping of woodland strawberry (Fragaria vesca) with oligopaint FISH probes. Cytogenetic Genome Res.

[41] Braz GT, He L, Zhao H, Zhang T, Semrau K, Rouillard JM (2018). Comparative oligo-FISH mapping: an efficient and powerful methodology to reveal karyotypic and chromosomal evolution. Genetics.

[42] Fields BD, Nguyen SC, Nir G, Kennedy S (2019). A multiplexed DNA FISH strategy for assessing genome architecture in Caenorhabditis elegans. eLife.

[43] Boettiger AN, Bintu B, Moffitt JR, Wang S, Beliveau BJ, Fudenberg G (2016). Super-resolution imaging reveals distinct chromatin folding for different epigenetic states. Nature.

[44] Šimoníková D, Němečková A, Karafiátová M, Uwimana B, Swennen R, Doležel J (2019). Chromosome painting facilitates anchoring reference genome sequence to chromosomes in situ and integrated karyotyping in banana (Musa spp.). Front Plant Sci.

[45] Gelali E, Girelli G, Matsumoto M, Wernersson E, Custodio J, Mota A (2019). iFISH is a publically available resource enabling versatile DNA FISH to study genome architecture. Nat Commun.

[46] Coombe L, Li JX, Lo T, Wong J, Nikolic V, Warren RL (2021). LongStitch: high-quality genome assembly correction and scaffolding using long reads. BMC Bioinformatics.

[47] Bonnet A (2001). Cytogenetic comparison between Vietnamese sika deer and cattle: R-banded karyotypes and FISH mapping. Chromosom Res.

[48] Damas J, O’Connor R, Farré M, Lenis VPE, Martell HJ, Mandawala A (2017). Upgrading short-read animal genome assemblies to chromosome level using comparative genomics and a universal probe set. Genome Res.

[49] Frohlich J, Kubickova S, Musilova P, Cernohorska H, Muskova H, Vodicka R (2017). Karyotype relationships among selected deer species and cattle revealed by bovine FISH probes. PLoS ONE.

[50] Joseph S, O’Connor R, Al Mutery A, Watson M, Larkin D, Griffin D (2018). Chromosome level genome assembly and comparative genomics between three falcon species reveals an unusual pattern of genome organisation. Diversity.

[51] Farré M, Li Q, Darolti I, Zhou Y, Damas J, Proskuryakova AA (2019). An integrated chromosome-scale genome assembly of the Masai giraffe (Giraffa camelopardalis tippelskirchi). GigaScience.

[52] Nurk S, Koren S, Rhie A, Rautiainen M, Bzikadze AV, Mikheenko A (2022). The complete sequence of a human genome. Science.

[53] National Library of Medicine. GRCm39 - mm39 - Genome - Assembly - NCBI. 2022. https://www-ncbi-nlm-nih-gov.acces.bibl.ulaval.ca/assembly/GCF_000001635.27. Accessed 20 June 2022.

[54] Bracewell R, Tran A, Chatla K, Bachtrog D (2020). Chromosome-level assembly of Drosophila bifasciata reveals important karyotypic transition of the X chromosome. G3.

[55] National Library of Medicine (2022). GRCz11 - danRer11 - Genome - Assembly – NCBI.

[56] Li M, Sun C, Xu N, Bian P, Tian X, Wang X (2022). De novo sssembly of 20 chicken genomes reveals the undetectable phenomenon for thousands of core genes on microchromosomes and subtelomeric regions. Mol Biol Evol.

[57] Rosen BD, Bickhart DM, Schnabel RD, Koren S, Elsik CG, Tseng E (2020). De novo assembly of the cattle reference genome with single-molecule sequencing. Gigascience.

[58] Warr A, Affara N, Aken B, Beiki H, Bickhart DM, Billis K (2020). An improved pig reference genome sequence to enable pig genetics and genomics research. GigaScience.

[59] Qiao G, Xu P, Guo T, Wu Y, Lu X, Zhang Q (2022). Genetic basis of dorper sheep (Ovis aries) revealed by long-read de novo genome assembly. Front Genet.

[60] Li R, Yang P, Dai X, Asadollahpour Nanaei H, Fang W, Yang Z (2021). A near complete genome for goat genetic and genomic research. Genet Sel Evol.

[61] Totikov A, Tomarovsky A, Prokopov D, Yakupova A, Bulyonkova T, Derezanin L (2021). Chromosome-level genome assemblies expand capabilities of genomics for conservation biology. Genes (Basel).

[62] Masabanda JS, Burt DW, O’Brien PCM, Vignal A, Fillon V, Walsh PS (2004). Molecular cytogenetic definition of the chicken genome: the first complete avian karyotype. Genetics.

[63] Roohi J, Cammer M, Montagna C, Hatchwell E (2008). An improved method for generating BAC DNA suitable for FISH. Cytogenetic Genome Res.

[64] Nguyen SC, Joyce EF (2019). Programmable chromosome painting with Oligopaints. Methods Mol Biol.

[65] Rogan PK, Cazcarro PM, Knoll JHM (2001). Sequence-based design of single-copy genomic DNA probes for fluorescence in situ hybridization. Genome Res.

[66] Graphodatsky AS, Perelman PL, O’Brien SJ (2020). Atlas of mammalian chromosomes.

[67] O’Brien SJ, Menninger JC, Nash WG (2006). Atlas of mammalian chromosomes.

[68] Proskuryakova AA, Ivanova ES, Perelman PL, Ferguson-Smith MA, Yang F, Okhlopkov IM, et al. Comparative studies of karyotypes in the Cervidae Family. Cytogenetic Genome Res. 2022;1–11. 10.1159/000527349. 10.1159/000527349 36463851

[69] Balmus G, Trifonov VA, Biltueva LS, O’Brien PCM, Alkalaeva ES, Fu B (2007). Cross-species chromosome painting among camel, cattle, pig and human: further insights into the putative Cetartiodactyla ancestral karyotype. Chromosom Res.

[70] Bana NÁ, Nyiri A, Nagy J, Frank K, Nagy T, Stéger V (2018). The red deer Cervus elaphus genome CerEla1.0: sequencing, annotating, genes, and chromosomes. Mol Gen Genomics.

[71] Neitzel H (1987). Chromosome evolution of cervidae: Karyotypic and molecular aspects. Cytogenetics : basic and applied aspects.

[72] Prakash B, Kuosku V, Olsaker I, Gustavsson I, Chowdhary BP (1996). Comparative FISH mapping of bovine cosmids to reindeer chromosomes demonstrates conservation of the X-chromosome. Chromosom Res.

[73] Bonnet-Garnier A, Claro F, Thévenon S, Gautier M, Hayes H (2003). Identification by R-banding and FISH of chromosome arms involved in Robertsonian translocations in several deer species. Chromosom Res.

[74] Chi J, Fu B, Nie W, Wang J, Graphodatsky AS, Yang F (2005). New insights into the karyotypic relationships of Chinese muntjac *(Muntiacus reevesi)*, forest musk deer *(Moschus berezovskii)* and gayal *(Bos frontalis)*. Cytogenetic Genome Res.

[75] Dementyeva PV, Trifonov VA, Kulemzina AI, Graphodatsky AS (2010). Reconstruction of the putative cervidae ancestral karyotype by chromosome painting of siberian roe deer (Capreolus pygargus) with dromedary probes. Cytogenetic Genome Res.

[76] Xu Q, Schlabach MR, Hannon GJ, Elledge SJ (2009). Design of 240,000 orthogonal 25mer DNA barcode probes. Proc Natl Acad Sci.

[77] Crosetto N, Bienko M, Gelali E, Girelli G, Matsumoto M, Wernersson E, et al. iFISH:a publically available resource enabling versatile DNA FISH to study genome architecture. Protocol Exchange. 2019; https://protocolexchange.researchsquare.com/article/nprot-7403/v1. Accessed 19 Sept 2020. 10.1038/s41467-019-09616-w PMC6456570 30967549

[78] Howe B, Umrigar A, Tsien F (2014). Chromosome preparation from cultured cells. J Vis Exp.

[79] Schindelin J, Arganda-Carreras I, Frise E, Kaynig V, Longair M, Pietzsch T (2012). Fiji: an open-source platform for biological-image analysis. Nat Methods.

[80] Gandrud C (2022). D3 JavaScript network graphs from R.

[81] Li H (2022). Minimap2.

[82] Chu J (2022). Circos assembly consistency (Jupiter) plot.

