# Supplementary figures and images for: Chromosome-level assembly of the Rangifer tarandus genome and validation of cervid and bovid evolution insights
Source: BMC Genomics. 2023 Mar 23;24:142. doi: 10.1186/s12864-023-09189-5 (PMC10037892; doi:10.1186/s12864-023-09189-5)

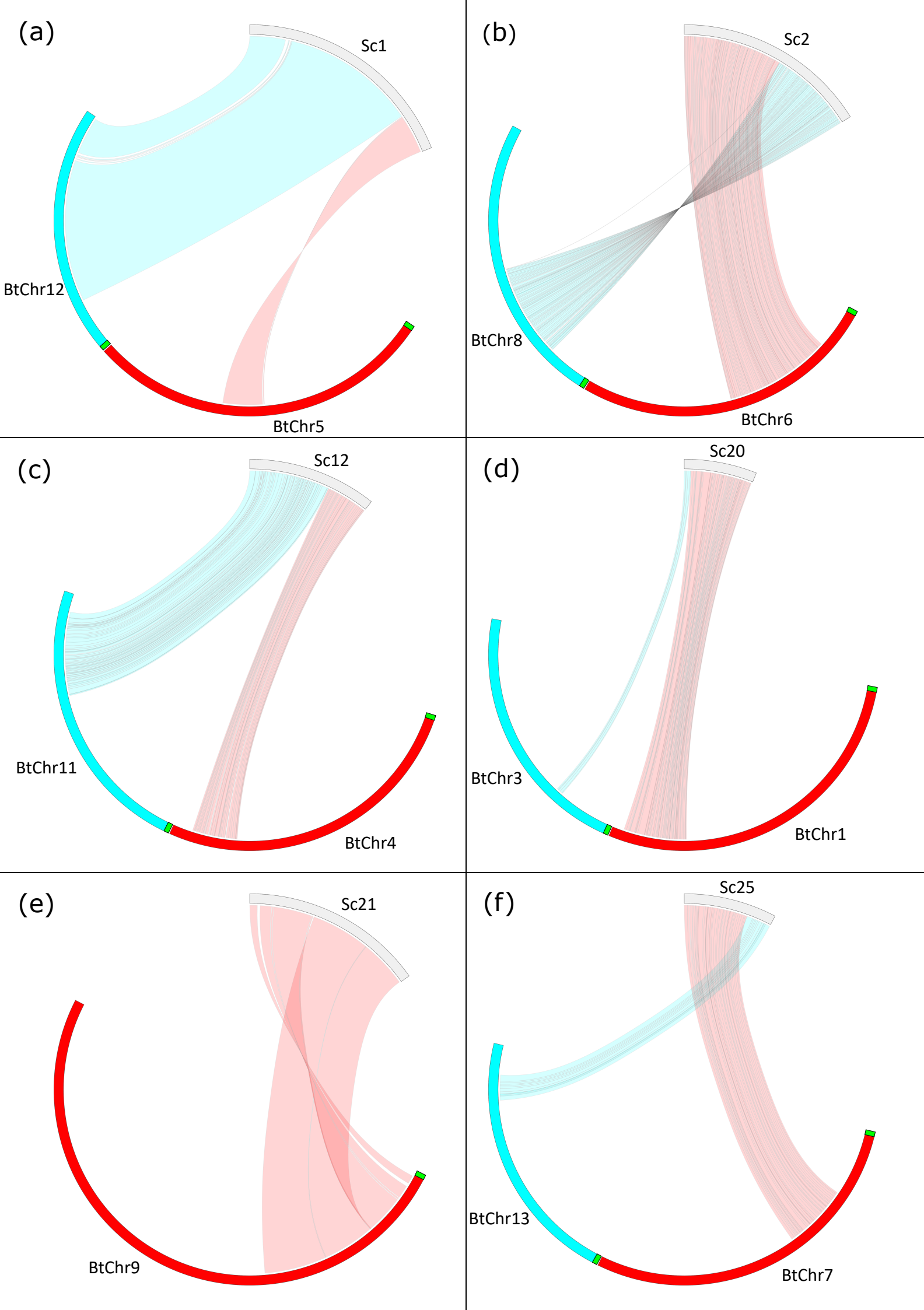

Supplement: Supplementary file 5 — Additional file 5: Fig. S1. Rangifer tarandus scaffold mis-assembly revealed by comparison with Bos taurus chromosomes. (a) Scaffold 1 mapped to bovine chromosomes 12 and 5; (b) Scaffold 2 mapped to bovine chromosomes 8 and 6; (c) Scaffold 12 mapped to bovine chromosomes 11 and 4; (d) Scaffold 20 mapped to bovine chromosomes 3 and 1; (e) Rearranged scaffold 21 mapped to bovine chromosome 9; (f) Scaffold 25 mapped to bovine chromosomes 13 and 7. Exact breakpoints are listed in Table S1. Green rectangles represent centromere positions. [file 12864_2023_9189_MOESM5_ESM.pdf]

BtChr26

Sc18

Sc14

BtChr28

Sc68

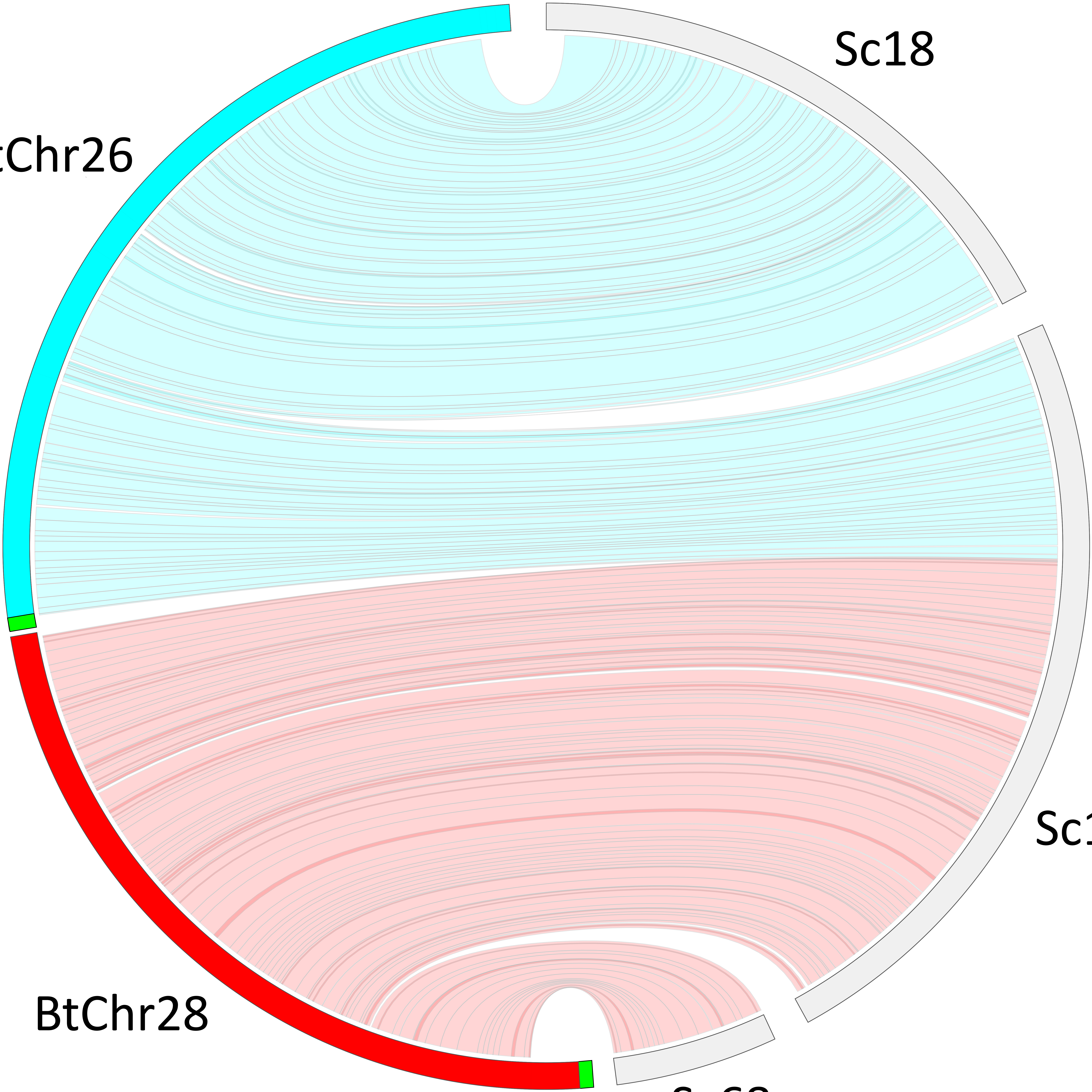

Supplement: Supplementary file 6 — Additional file 6: Fig. S2. Scaffolds 18, 14 and 68 (top to bottom order) forming R. tarandus chromosome 6 and corresponding to split bovine chromosomes 26 and 28. The fission that led to the new chromosomes has been carried in scaffold 14. The centromere of R. tarandus chromosome 6 apparently has been conserved in bovine chromosome 28 based on genome mapping. Green rectangles represent centromere positions. [file 12864_2023_9189_MOESM6_ESM.pdf]

BtChr1

RtChr34

RtChr27

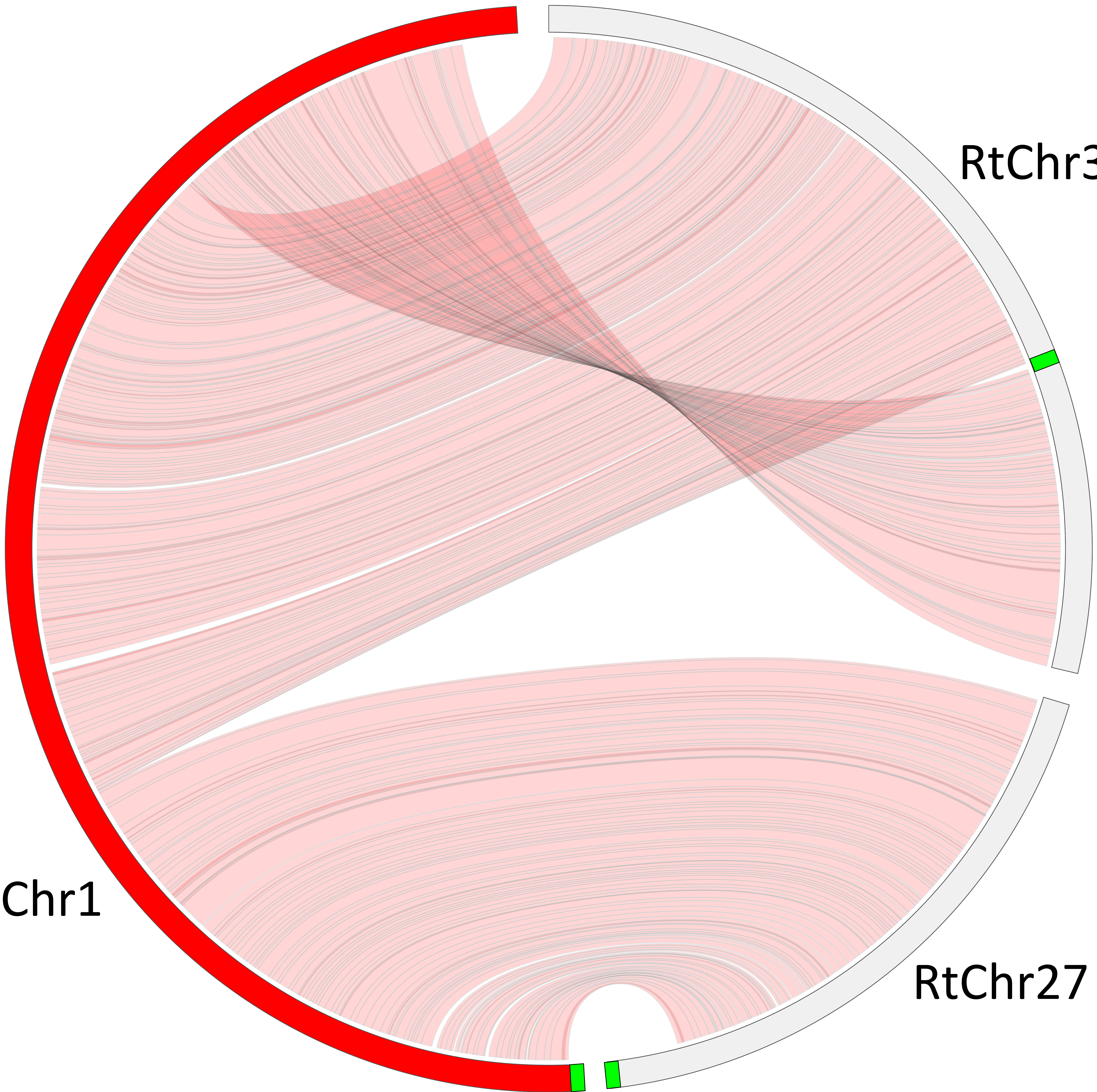

Supplement: Supplementary file 7 — Additional file 7: Fig. S3. Mapping of chromosomes 27 and 34 as assembled in this study on bovine chromosome 1 . Chromosomal fission led to the formation of the two R. tarandus chromosomes. The distal portion of the larger chromosome subsequently underwent translocation and inversion, the latter rearrangement creating the submetacentric character of R. tarandus chromosome 34. Green rectangles represent centromere positions. [file 12864_2023_9189_MOESM7_ESM.pdf]
