# Supplementary material for: Chromosome-level assembly of the Rangifer tarandus genome and validation of cervid and bovid evolution insights
Source: BMC Genomics. 2023 Mar 23;24:142. doi: 10.1186/s12864-023-09189-5 (PMC10037892; doi:10.1186/s12864-023-09189-5)
Supplement: Supplementary file 8 — Additional file 8: Table S1. Breakage positions in the mis-assembled scaffolds. [file 12864_2023_9189_MOESM8_ESM.docx]

Table S1. Breakage positions in the mis-assembled scaffolds

| Scaffold | Bovine chromosomal homolog | Break point on scaffold (nt) | Probe before | | Probe after | |
| --- | --- | --- | --- | --- | --- | --- |
|  |  |  | **ID** | **Position (nt)** | **ID** | **Position (nt)** |
| 1 | 5, 12 | 64437838 | P1.2 | 54997421 | P2 | 64864627 |
| 2 | 6, 8 | 34711592 | P1 | 29811875 | P2 | 48020758 |
| 12 | 4, 11 | 16590016 | P0 | 3712536 | P1 | 16941533 |
| 20 | 1, 3 | 30264322 | P1 | 18516306 | P2 | 30665740 |
| 21 | 9 | 11606985 | P0 | 2870441 | P1 | 14352306 |
| 25 | 7, 13 | 8363321 | P0 | 2524222 | P1 | 13372385 |

Probe before = designed probe before the break point; Probe after = designed probe after the break point
